# An update: epigenetic mechanisms underlying methamphetamine addiction

**DOI:** 10.3389/fcell.2024.1494557

**Published:** 2024-11-22

**Authors:** Mingxin Liu, Zizhen Si

**Affiliations:** Department of Medicine, Ningbo University, Ningbo, Zhejiang, China

**Keywords:** epigenetics, methamphetamine, DNA methylation, histone modification, non-coding RNAs

## Abstract

Methamphetamine (METH) is one of the most widely abused illicit drugs globally. Despite its widespread abuse, the effects of methamphetamine on the brain and the precise mechanisms underlying addiction remain poorly understood. Elucidating these biological mechanisms and developing effective treatments is of utmost importance. Researchers have adopted a multi-faceted approach, combining studies at the genetic, molecular, organ, and individual levels, to explore the epigenetic changes that methamphetamine use brings to an organism from both micro and macro perspectives. They utilize a comparative analysis of experimental animal data and clinical cases to ascertain differences and identify potential targets for translating METH addiction research from the experimental to the clinical setting. Recent studies have demonstrated that epigenetic regulation plays a pivotal role in neural mechanisms, encompassing DNA methylation, histone modifications (such as acetylation and methylation), ubiquitination, phosphorylation, and the regulation of non-coding RNA. These epigenetic factors influence an individual’s susceptibility and response to methamphetamine addiction by regulating the expression of specific genes. Specifically, methamphetamine use has been observed to cause alterations in DNA methylation status, which in turn affects the expression of genes associated with neuroreward pathways, leading to alterations in brain function and structure. Furthermore, histone modifications have significant implications for the neurotoxicity associated with methamphetamine addiction. For instance, the methylation and acetylation of histone H3 modify chromatin structure, consequently influencing the transcriptional activity of genes. Non-coding RNAs, including microRNAs (miRNAs) and long non-coding RNAs (lncRNAs), also play a pivotal role in methamphetamine addiction by interacting with messenger RNAs (mRNAs) and regulating gene expression. To further advance our understanding, researchers employ advanced technologies such as high-throughput sequencing, chromatin immunoprecipitation sequencing (ChIP-seq), and RNA sequencing (RNA-seq) to comprehensively analyze epigenetic changes in both animal models and human subjects. These technologies enable researchers to identify specific epigenetic markers associated with methamphetamine addiction and to explore their functional consequences. This article reviews the role of these epigenetic mechanisms in methamphetamine addiction and discusses their potential implications for future clinical treatment strategies, particularly in the development of drugs targeting methamphetamine addiction. By deepening our comprehension of these epigenetic regulatory mechanisms, it is anticipated that targeted therapeutic strategies may be devised to reverse the gene expression alterations associated with methamphetamine addiction, thus enhancing the efficacy of addiction treatment and paving the way for future research in this domain.

## 1 Epigenetic mechanisms

Since Conrad Waddington first coined the term “epigenetics” in 1942, research has progressively unveiled the intricate mechanisms through which organisms produce potentially heritable and non-heritable variation in response to environmental factors, without altering the DNA sequence (Villasenor and Baubec, 2021; Bhat et al., 2021). Epigenetics involves the study of changes in gene expression that occur independently of structural changes in the DNA sequence. These modifications enable organisms to adapt to environmental stimuli, resulting in alterations in gene expression that can be propagated through processes such as DNA methylation, histone modification, and the regulation of non-coding RNAs. Such epigenetic alterations endow the same DNA sequences with diverse functionalities under varying conditions, thereby significantly influencing the organism’s behavior, learning, memory, and cognitive functions. For example, DNA methylation, a key epigenetic modification, plays a crucial role in the development and function of the nervous system by affecting chromatin structure and gene transcriptional activity (Jones, 2012).

**FIGURE 1:**
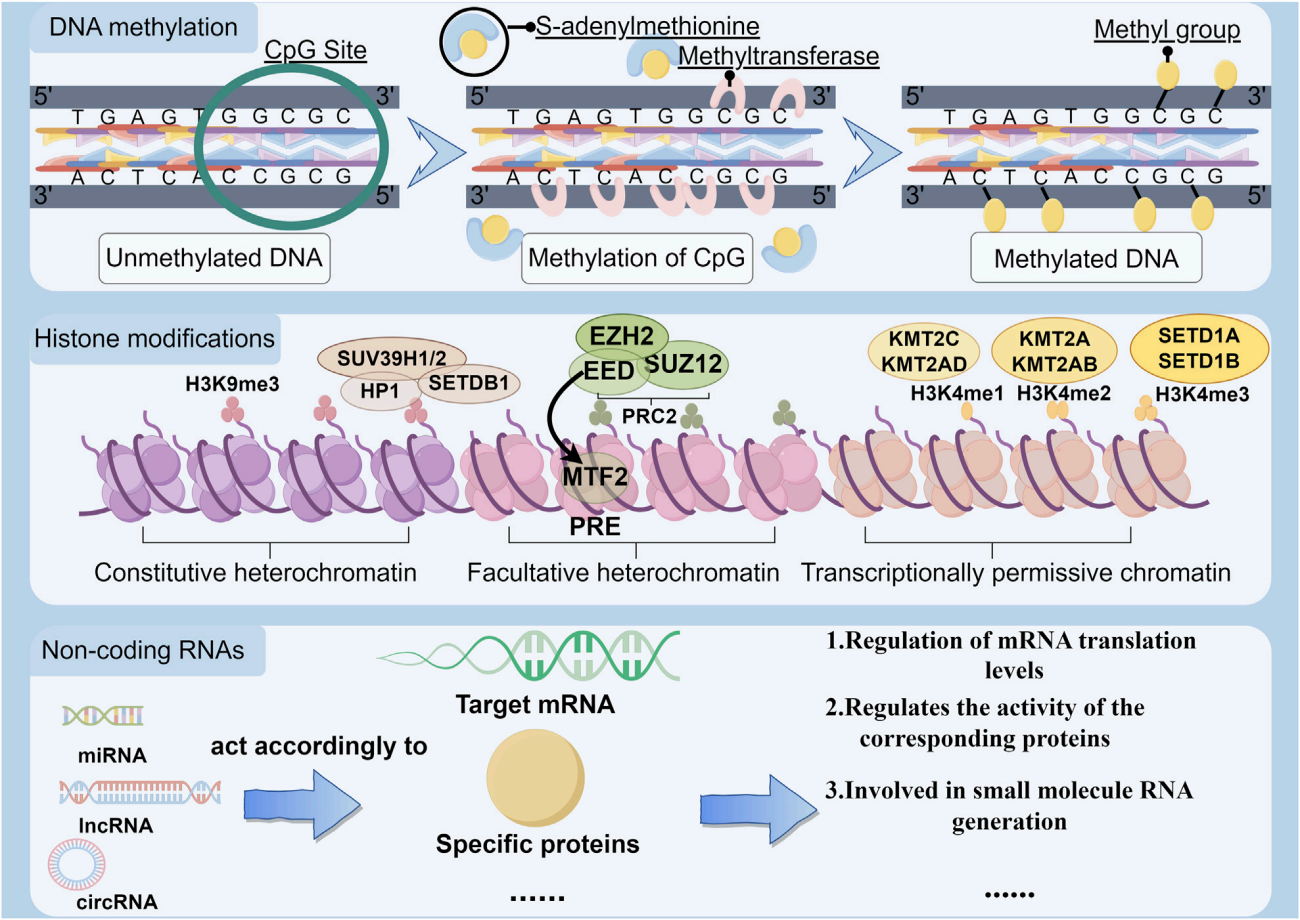
Three major mechanisms of epigenetic modification.The diagram illustrates, by region, the molecular process through which DNA is methylated by the addition of methyl groups specifically on CpG islands. Additionally, it delineates the gene loci on exposed chromatin where histone modifications play a crucial role, and it further elucidates the various ways in which different types of non-coding RNA modifications are epigenetically active.

Epigenetic effects are crucial in maintaining diverse, cell-specific gene expression profiles. By regulating gene expression within homeostatic ranges, epigenetic modifications are able to elicit sustained responses of relevant genes to specific stimuli. These responses lead to altered cellular phenotypes, which may scale up to cause structural and functional abnormalities at the organismal level (Feil and Fraga, 2012). Epigenetic mechanisms are fundamental in the development of addiction-related neuromorphological changes, such as alterations in neuron structure and connectivity (Cadet and Jayanthi, 2021). Addictive behaviors often lead to long-term structural and functional adaptations in the nervous system, predominantly through epigenetic modifications like DNA methylation and histone acetylation. These changes contribute to the persistence of addictive states and behaviors (Zeng et al., 2023) (Figure 1).

## 2 The role of methamphetamine in relevant brain regions

Methamphetamine (METH), a potent central nervous system (CNS) stimulant, has evolved into a major global public health challenge since the late 1990s (Hong et al., 2021). METH abuse is not only associated with a wide range of cognitive deficits, but also has significant neurobehavioural impacts, including aggression and psychotic symptoms (Han et al., 2021). These are attributed to METH’s significant neurotoxicity to the CNS. In the context of METH use disorders, the reward circuit plays a central role. This circuit involves multiple brain subregions, including the ventral tegmental area (VTA), nucleus accumbens (NAc), dorsal striatum (DS), amygdala (AMG), hippocampus (Hip), and prefrontal cortex regions (PFC) (Zhang et al., 2021; Rezayof et al., 2023). In these key regions, METH is able to trigger significant transcriptional and epigenetic changes, which in turn produce complex biological effects.

**FIGURE 2:**
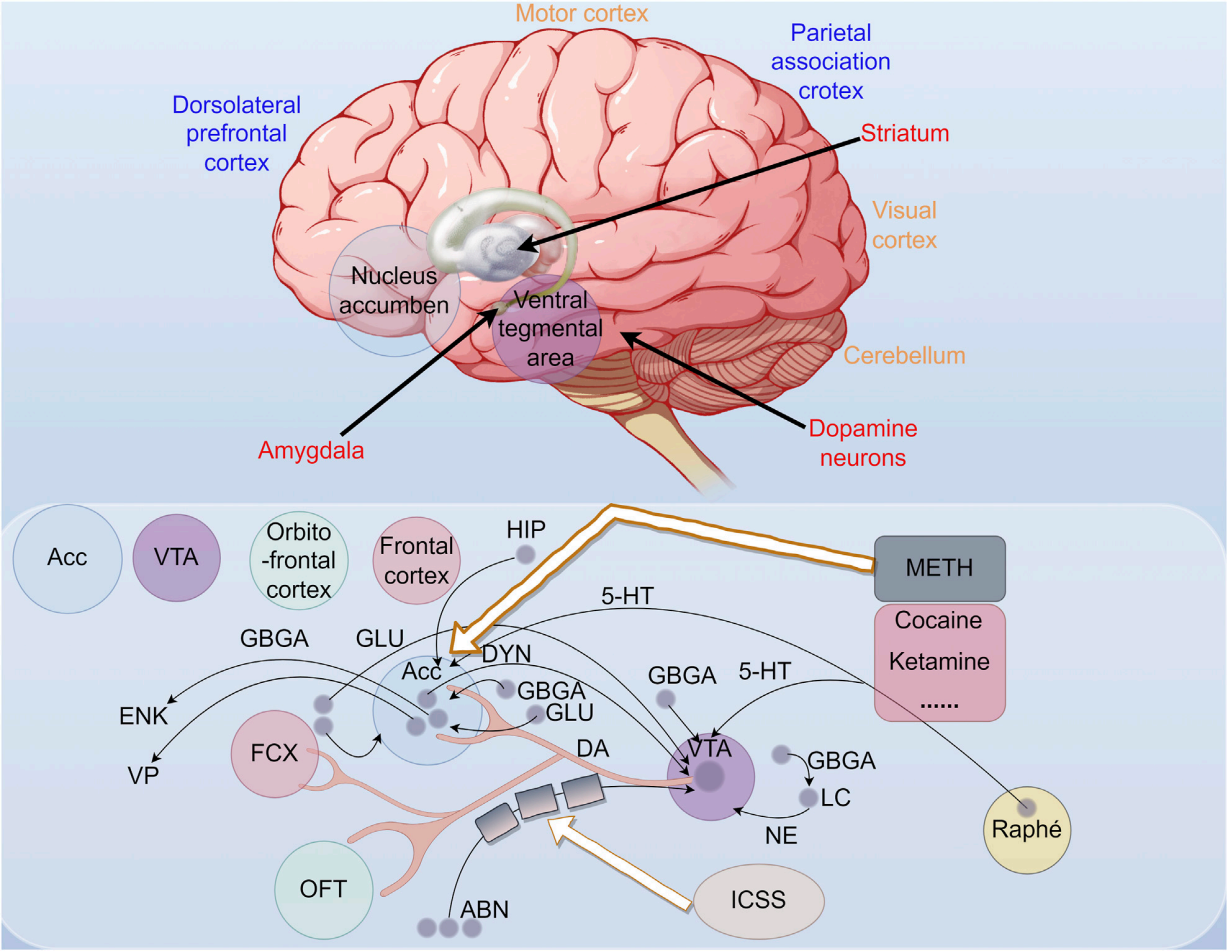
The red structure represents the primary neuronal groups that encode rewards without sensory stimulation or motor action. The blue structure represents neuronal groups that encode rewards with sensory stimuli or motor actions, and the orange structure represents non-reward coding neuronal groups. The diagram illustrates the brain’s reward circuitry activated by addictive drugs, leading to behaviors such as drug-seeking, consumption, cravings, and relapse. The nucleus ambiguus is a key component, which regulates physiological functions and is part of the dopaminergic system. Endogenous opioid peptides, dynorphin and enkephalin, play significant roles in pain perception, emotional responses, and reward systems. Dynorphin is the ligand for the κ-opioid receptor, and enkephalin is for the δ-opioid receptor. The prefrontal cortex is also crucial. GABA is a major inhibitory neurotransmitter, while glutamate maintains the excitation-inhibition balance. Abnormal glutamate activity is linked to neurodevelopmental disorders. Glutamic acid, which glutamate is derived from, acts as an excitatory neurotransmitter. 5-HT (serotonin) regulates mood, sleep, appetite, cognitive function, and pain perception. ICSS studies reward behaviors through electrical brain stimulation. The locus coeruleus (LC), involved in stress, attention, and emotion regulation, interacts with the dopamine system in the VTA, influencing reward and addiction. The Raphe nuclei are serotonergic, and the VTA is key in reward processing and addiction.

The midbrain limbic dopamine (DA) circuit plays a pivotal role in the central ”reward system”. The circuit originates from dopaminergic neuronal somata specifically in the VTA of the midbrain. Fibers from the VTA project to the limbic system and cortex via the medial forebrain bundle, terminating in the NAc. The abuse of addictive drugs results in pathological alterations in the neural circuits and nuclei associated with the reward system, with a notable increase in the function of midbrain dopamine neurons, particularly in the area of the NAc (Ross and Peselow, 2009; Lammel et al., 2014). The NAc, an independent brain structure implicated in emotional and reward processes, may undergo alterations in gene expression following exposure to METH. These changes could potentially influence the release and reception of dopamine, thereby enhancing addictive behaviors. The dentate gyrus, a component of the hippocampus, is known for its involvement in memory and learning processes. METH exposure has the potential to influence genes associated with neuroplasticity and memory formation in this region, which may result in altered cognitive function. The Cornu Ammonis (CA) region of the hippocampus is subdivided into distinct subregions, such as CA1 and CA3, and METH exposure may affect gene expression in these subregions, potentially impacting neuronal excitability and synaptic plasticity. Additionally, the subventricular zone (SVZ), the primary source of neural stem cells, may experience alterations in the proliferation and differentiation of these cells due to METH exposure, subsequently influencing the brain’s capacity for repair and regeneration (Miao et al., 2023) (Figure 2).

METH enters neurons and nerve endings through its action on DA transporter proteins (DATs) on presynaptic membranes, displacing DA in the cytoplasm and vesicles. Furthermore, METH was found to affect the function of DAT on the plasma membrane, inducing its redistribution, which resulted in a massive efflux of DA from the cytoplasm into the synaptic gap (Zahniser and Sorkin, 2009; Kahlig et al., 2006). This, in turn, enhanced the function of midbrain DA neurons. METH also acts on the type II monoamine vesicular transporter (VMAT-2) on DA neuron vesicles, preventing DA synthesised in the cytoplasm from entering the vesicles for storage by interfering with its function and inducing the release of DA from the vesicles into the cytoplasm. These DAs are converted to reactive oxides (ROS) by monoamine oxidase enzymes and auto-oxidation (Eyerman and Yamamoto, 2007). These ROS interact with H₂O₂ and NO to produce the next level products of ROS, which, together with elevated concentrations of DA in the synaptic gap, ultimately lead to the onset of dopaminergic neurotoxicity (Shin et al., 2021).

The 5-hydroxytryptamine (5-HT) system is also a significant factor in the process of METH addiction. Although 5-HT does not directly cause neurotoxicity to neurons, it acts indirectly by regulating the release of DA in the central ‘reward system’ (Thomas et al., 2010). Two mechanisms are involved in the effect of METH on 5-HT. Firstly, 5-HT levels are increased in the brain through the function of the 5-HT reuptake transporter (SERT), which METH inhibits (Zhang et al., 2021; Scearce-Levie et al., 1999). Secondly, METH induces the release of large quantities of 5-HT into the synaptic cleft. A significant amount of endogenous 5-HT is capable of increasing DA synthesis and release (Zhang et al., 2021). The study by Proudnikov et al. further demonstrated that direct injection of 5-HT3 receptor agonists into the NAc was able to elevate the levels of DA in this region, whereas 5-HT3 receptor antagonists were able to inhibit the bioelectrical activity of the elevated DA in the NAc (Proudnikov et al., 2006).

**FIGURE 3:**
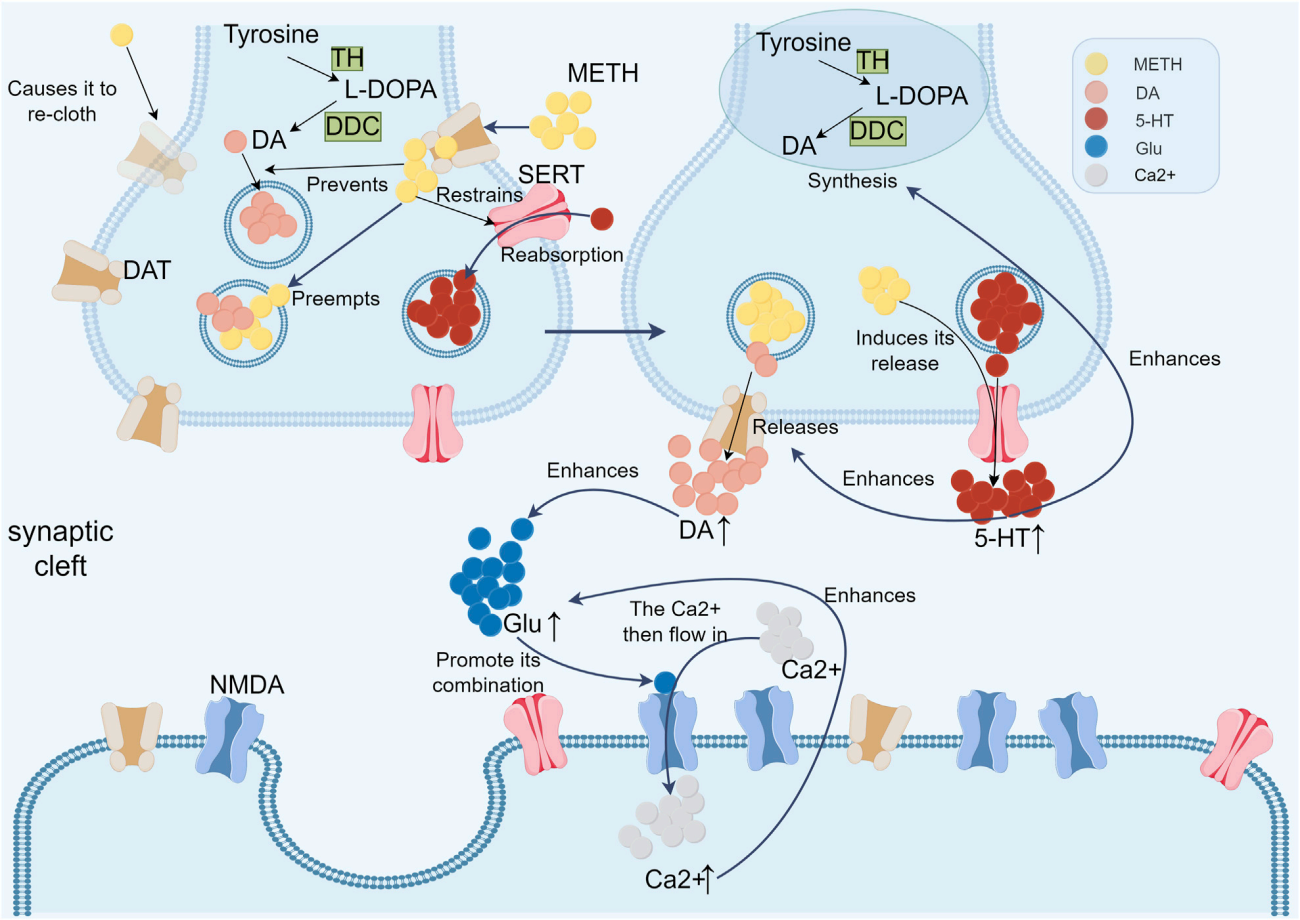
The following flow chart illustrates the action of three transmitters (DA, 5-HT and Glu) in the context of methamphetamine addiction. METH enters neurons through DATs on presynaptic membranes, displacing DA in the cytoplasm and vesicles, causing a massive DA efflux into the synaptic gap, enhancing midbrain DA neuron activity. It also acts on VMAT-2 on DA vesicles, preventing DA storage and inducing DA release into the cytoplasm, where it is converted to ROS. These ROS interact with H₂O₂ and NO, leading to dopaminergic neurotoxicity. Additionally, METH indirectly affects the 5-HT system by regulating DA release in the central reward system. METH inhibits the SERT, increasing 5-HT levels and inducing its release into the synaptic cleft, boosting DA synthesis and release. Studies show that 5-HT3 receptor agonists elevate DA levels in the NAc, while antagonists inhibit this activity. Furthermore, METH increases extracellular Glu concentration by promoting abnormal DA release. Glu activates NMDA receptors, causing high neuronal excitation and neurotoxicity. This activation leads to a sustained inward Ca2+ flow, increasing intracellular Ca2+ levels, further stimulating Glu release, disrupting Ca2+ homeostasis, and perpetuating neurotoxicity.

The potential mechanism of METH toxicity to the nervous system was further elucidated in a study by (Fleckenstein et al., 2007). The researchers demonstrated that METH can increase extracellular glutamate (Glu) concentration by promoting abnormal DA release. Glu, an excitatory amino acid, activates the N-methyl-D-aspartate (NMDA) receptor, leading to high neuronal excitation and ultimately neurotoxicity. The activation of the NMDA receptor causes a slow and sustained inward flow of Ca^2+^, resulting in a significant increase in the intracellular Ca^2+^ level. This elevated intracellular Ca^2+^ concentration in turn stimulates further Glu release, thereby establishing a vicious cycle that results in a continuous increase in intracellular Ca^2+^ levels and ultimately disrupts intracellular Ca^2+^ homeostasis (Schinder et al., 1996) (Figure 3).

## 3 Methamphetamine addiction and DNA methylation

In the 1980s, the regulatory role of DNA methylation in gene expression and cell differentiation gradually received widespread attention. Although DNA methylation does not directly affect base pairing, it can significantly influence DNA-protein interactions, which in turn has a profound effect on gene expression (Compere and Palmiter, 1981; Kirtana et al., 2020).

TET proteins facilitate the oxidation of 5-methylcytosine (5 mC) to 5-hydroxymethylcytosine (5hmC), which is subsequently oxidised to 5-formylcytosine (5 fC) and then to 5-carboxycytosine (5caC) (Liang et al., 2024). Ultimately, this process leads to the removal of methylation through the base excision repair pathway. During DNA replication, 5hmC impairs the ability of DNA methyltransferase (DNMT1) to recognize and methylate cytosine on newly synthesized strands, consequently reducing overall methylation levels. The decrease in DNA methylation levels, achieved through either of these two processes, enhances chromatin openness. Typically, 5hmC is concentrated in the promoter and enhancer regions of genes, serving as a pivotal regulatory site for gene transcription. The resultant open chromatin structure provides an optimal environment for the binding of transcription factors and RNA polymerase, thereby facilitating gene transcription and expression (Liang et al., 2024). Brain-derived neurotrophic factor (BDNF) plays a pivotal role in neuronal survival, differentiation, and synaptic plasticity, and is instrumental in promoting neuronal growth and function. Tropomyosin receptor kinase B (TrkB), as a receptor for BDNF, is involved in neuronal survival and differentiation. Neurogenin 1 (Ngn1) is an essential transcription factor in neuronal differentiation, regulating neuron generation and differentiation processes. Paired box 6 (PAX6) plays a crucial role in neuronal differentiation and brain development, affecting neuronal development and maturation. Arc plays a pivotal role in synaptic plasticity and long-term potentiation (LTP), regulating synaptic function in neurons. Syn1 is a crucial regulator of synaptic vesicle cycling and neurotransmitter release, affecting nerve conduction. Glial cell line-derived neurotrophic factor (GDNF) plays a vital role in neuronal survival and repair, promoting neuronal growth and regeneration. 5hmC facilitates the transcription and expression of these genes by enriching for the regions that are required to regulate promoters or enhancers. This process, facilitated by 5hmC, promotes the transcription and expression of these genes, thereby regulating and influencing neuronal formation and function. The following section will focus on the genes and effects regulated by 5hmC in immediate neuronal function. In comparison to methamphetamine-addicted rats, 5hmC has been observed to induce significant differential hydroxymethylation in the nucleus accumbens (NAc) of non-addicted rats, accompanied by an increase in mRNA levels, particularly in genes encoding voltage-gated potassium channels such as Kv1.2, Kvbeta1, and Kv2.2, as well as the calcium-gated potassium channel genes KCNMA1, KCNN1, and KCNN2 (Cadet et al., 2017). These genes play a significant role in regulating neuronal excitability, neurotransmitter release, and synaptic plasticity.

The maintenance of DNA methylation is dependent on specific methyltransferases (DNMTs), particularly those such as DNMT3A and DNMT3B, which play a crucial role in the regulation of DNA methylation (Li and Zhang, 2014). It has been demonstrated that METH is capable of affecting the expression of DNA methyltransferases and inducing changes in DNA methylation that play a pivotal role in cognitive learning and memory (Miao et al., 2023). For instance, acute and chronic METH injections were found to increase the expression of DNMT1 and DNMT3 in the NAc and DS of rats (Jayanthi et al., 2020; Numachi et al., 2007). DNMT3A, a DNA methyltransferase that functions specifically in neurons, is responsible for adding methyl groups to the cytosine (C) bases of DNA, thereby forming 5-methylcytosine (Laplant et al., 2010). This modification typically leads to the repression of gene expression. An increase in DNA methylation due to DNMT3A activity can suppress genes related to neuroplasticity and reward mechanisms, encompassing those that regulate neurotransmitter release and synaptic plasticity. Chronic methamphetamine use has been shown to elevate DNMT3A activity, which subsequently increases the density of dendritic spines in NAc neurons. These dendritic spines serve as the connection points between neurons. Consequently, an increase in their density enhances neuronal signaling. The morphology of neuronal dendritic spines can also change, manifesting as elongated, mushroom-shaped, or short and thick forms (Yap et al., 2021). These changes are linked to enhanced synaptic function and are associated with neuronal connections and signaling that can promote addictive behavior. DNMT3A expression remains upregulated even after prolonged withdrawal, indicating its crucial role in maintaining the addictive state. This persistent modulation of gene expression may result in long-term behavioral and neurological alterations. Furthermore, METH self-administration was demonstrated to elevate DNA methylation levels of several potassium channel genes in the rat brain (Jayanthi et al., 2020). It is notable that the withdrawal period (e.g., 30 days) following acute METH injection leads to an increase in mRNA expression of the stress-related genes Crh and Avp (Jayanthi et al., 2018). This is attributed to DNA hypomethylation of the CpG site near the promoter region of Crh and the intronic region of Avp. Conversely, chronic METH injection results in elevated protein levels of methylated CpG binding protein 2 (MECP2) and co-precipitation of MECP2 with HDAC2, which further inhibits the transcription of the AMPA glutamate receptor (Jayanthi et al., 2021). The findings were further substantiated by Chromatin Immunoprecipitation (ChIP) assay, which demonstrated that METH administration led to an increase in MECP2 enrichment in the GluA2 and GluA1 promoter regions (Jayanthi et al., 2021). Furthermore, Methylated CpG DNA Immunoprecipitation-Polymerase Chain Reaction (MeDIP-PCR) demonstrated a reduction in cytosine methylation at CpG sites situated in close proximity to the GluA2 promoter site and at a CpG-rich site located at −1 kb upstream of the GluA2/3 promoter region (Jayanthi et al., 2014). In addition to changes at the CpG site, which are known alterations in DNA, other alterations, such as Long Interspersed Nuclear Element-1 (LINE-1) activity, are also regulated by DNA methylation and histone modifications (Moszczynska, 2020). According to Moszczynska et al., METH injections exhibited increased LINE-1 activity in the dentate gyrus of the Hip and the DStr regions, which may contribute to METH-induced cognitive and memory deficits (Moszczynska, 2020).

In a study by Xinyu Fan et al. (2020), oxytocin (OT) was found to inhibit the alterations caused by METH addiction by modulating DNA methylation at the synaptophysin promoter. METH administration resulted in hypomethylation of the Syn promoter-associated CpG islands in the hippocampus (Hip) and hypermethylation of the same region in the prefrontal cortex (PFC) of mice. However, mice pretreated with OT demonstrated the ability to suppress the effects of METH on cognition and memory by increasing the methylation of Syn promoter-associated CpG islands in the hippocampus, while concomitantly decreasing the methylation of Syn promoter-associated CpG islands in the Hip and increasing the level of Syn mRNA specifically in the PFC (Godino et al., 2015). This resulted in the recovery of cognitive and memory functions in the mice to a degree comparable to that of the non-drugged controls. These findings indicate that METH affects the organism’s behavioural sensitivity level by specifically regulating DNA methylation at the synaptophysin promoter in distinct regions. Furthermore, OT treatment may represent a promising therapeutic strategy for the cessation of METH addiction.

In a clinical study, the methylation levels of male METH users were examined using the Methylation-Sensitive LightCycler Real-time PCR (MSLR-PCR) technique. A significant correlation was found between the methylation level of chimerin 2 (CHN2) and METH dependence (Hao et al., 2017). CHN2 is a protein involved in actin cytoskeleton remodelling and Hip axon pruning, suggesting that chronic METH abuse may induce aberrant methylation of the CHN2 gene, which in turn interferes with actin cytoskeleton remodelling, leading to irregular neural protrusions and growth cones (Jayanthi et al., 2021). This is essential for sustaining persistent addictive behaviour. Apart from that, pyrophosphate sequencing studies have demonstrated a notable elevation in the methylation levels of petitin (PVALB) in the METH-dependent patient cohort, in contrast to the non-METH-dependent patient group, where no such change was observed. Alterations in the levels of PVALB may result in GABAergic deficits associated with METH dependence (Veerasakul et al., 2017). In individuals with a history of METH dependence, brain-derived neurotrophic factor (BDNF) functions as a molecular mediator, playing a pivotal role in the consolidation of memory in response to psychostimulant exposure (Rezayof et al., 2023; Xu et al., 2016 ^;^ Kim et al., 2020). Pyrophosphate sequencing of five CpG sites (CpG1-5) on the BDNF promoter revealed that only CpG5 methylation was significantly reduced in METH abusers. Decreased DNA methylation of BDNF exon IV led to decreased expression of BDNF, which plays an essential role in long-lasting potentiation and learning (Xu et al., 2016). Furthermore, reduced BDNF levels further induced impairment of cognitive and memory functions associated with METH addiction.

While understanding the widespread effects of methamphetamine (METH) on DNA methylation and gene expression is a significant step forward, the real challenge lies in determining which genetic changes are causal factors in the development of addiction and which are merely concomitant phenomena. It is therefore recommended that the temporal dynamics of the subject’s changes be analysed and that experiments be designed to capture these dynamics. Such experiments could, for example, compare gene expression and methylation status at different time points after METH exposure, including acute exposure, chronic exposure, and withdrawal. Furthermore, longitudinal studies are required to track molecular changes in individuals during the addiction process, with a particular focus on key nodes such as exposure, addiction, withdrawal and relapse. Furthermore, the functional validation of gene loci represents a crucial aspect of this process. Experiments for relevant validation by knocking out or knocking in specific genes through gene editing technology have already been conducted. Nevertheless, the transgenic mouse model, which involves manipulating gene expression at a specific point in time and verifying the function of these genes in the context of addiction, has not been widely employed. There is considerable scope for its application. This is not only in relation to validating METH on DNA methylation, but also in respect of the series of epigenetic roles of METH brain regions that are discussed in the following article.

## 4 Methamphetamine addiction and histone modification

Histone modification is a covalent modification that occurs mainly at amino acid residues in histone tails. These tails are modified by the addition of acetyl, methyl, phosphoryl, acyl, and ADP-ribosyl groups, among other relevant modifications (Baxter et al., 2014; Mottamal et al., 2015).

DARs (DNA reachable regions) are genomic regions that exhibit a more open chromatin structure, where histone modifications play a pivotal role in facilitating the binding of transcription factors and other regulatory proteins to DNA, thereby regulating gene expression. Modifications in chromatin accessible regions can indicate fluctuations in gene regulatory networks, especially in response to external stimuli, such as drug exposure. Observations show that 90% of DARs are located within intra- or intergenic regions, which indicates that distal regulatory elements (such as enhancers) within these regions are the principal targets of METH stimulation. Approximately 5% of DARs are located within the promoter regions of genes, and alterations in chromatin accessibility to these regions are positively correlated with gene expression (Miao et al., 2023). By identifying immediate homologous regions of DARs in the mouse genome and annotating these regions using ENCODE data, nearly 40% of DARs were identified as cis-regulators (such as enhancers) (Miao et al., 2023). These enhancers were classified as proximal and distal enhancers, with distal enhancers being associated with aberrant synaptic plasticity and playing an important role in methamphetamine addiction and the induction of neurotoxicity.

The majority of DARs have immediate homologous counterparts in the mouse and human genomes, indicating that these regions are highly conserved over evolutionary time. A greater than 70% conservation of DARs associated with methamphetamine (METH) exposure has been observed between rats and humans. This indicates that these regions have retained comparable gene regulatory functions throughout evolutionary history, with 24% exhibiting rodent-specific characteristics and 10% displaying rat-specific features. Genes situated in proximity to these conserved DARs are markedly enriched in essential brain functions, including brain development, neurogenesis, learning and memory (Miao et al., 2023). In contrast, genes located in the vicinity of rodent- and rat-specific DARs are more pertinent to histone modifications and immune responses. This indicates that the rat model exhibits a distinctive species-specific profile with regard to addiction research.

Despite evidence indicating that DARs (differentially accessible regions) are more than 70% conserved between rats and humans, suggesting that these regions have retained comparable gene regulatory functions throughout evolutionary history, it is notable that 24% of DARs are rodent-specific and 10% are rat-specific. This indicates that these regions have evolved species-specific characteristics. Therefore, when using rat models to study human addiction mechanisms, it is imperative to consider these species differences. It is plausible that rat-specific DARs are involved in gene regulatory mechanisms or responses that are unique to rats and may not be fully extrapolated to humans. Hence, when undertaking cross-species studies, it is crucial to exercise caution in interpreting functional differences and to consider the potential for species-specific implications. Notably, 90% of the aforementioned DARs are located within intragenic or intergenic regions, and these findings suggest that distal regulatory elements (e.g., enhancers) are the primary targets of METH (methamphetamine) stimulation. Distal regulatory elements are capable of influencing the expression of target genes through the 3D genomic structure over greater distances than proximal regulatory elements, which act directly on gene promoter regions. The complexity of this long-range regulation adds to the difficulty of studying these elements, as their actions frequently involve intricate spatial and temporal dynamics. To understand how these distal regulatory elements specifically regulate gene expression, more detailed functional validation and mechanistic studies are required. These include chromatin conformation capture technologies (e.g., Hi-C) to uncover the physical interactions between distal enhancers and their target genes, gene editing technologies (e.g., CRISPR-Cas9) to verify the functions and effects of these enhancers, and single-cell sequencing technologies to identify and elucidate the specific roles of distal regulatory elements in different cellular types.

### 4.1 Methamphetamine addiction and histone acetylation

In animal models of addiction, histone acetylation is a highly dynamic process of chromatin modification. The majority of modifications occur on specific lysine residues, such as K9 and K14 on H3 and K5, K8, K12, and K16 on H4 (Godino et al., 2015). The dynamic regulation of histone acetylation is mediated by the balance between the activities of histone acetyltransferases (HATs) and histone deacetylases (HDACs). Increased levels of histone acetylation are generally associated with increased gene expression, indicative of heightened transcriptional activity. Conversely, decreased levels of acetylation are linked to reduced gene expression levels. Under normal conditions, HATs and HDACs maintain a balanced homeostatic state in neurons (Wey et al., 2016). However, in neurodegenerative diseases, this equilibrium is often disrupted, leading to aberrant gene expression patterns (Nativio et al., 2020).

In a study conducted by Gonzalez et al. (2019). in 2019, the acute injection of METH (1 mg/kg) in mice resulted in increased levels of histone deacetylase 1 (HDAC1) and HDAC2, as well as pan-acetylated histone H3 proteins in the PFC, while pan-acetylated histone H4 protein was reduced (Martin et al., 2012). Conversely, in the NAc, HDAC1 protein levels were decreased, and HDAC2 protein levels were increased. These results correlated with a decrease in the abundance of acetylated histone H3 at lysine 9 (H3K9ac) and lysine 18 (H3K18ac), and an increase in the abundance of acetylated histone H4 at lysine 5 (H4K5ac) and lysine 8 (H4K8ac) (Martin et al., 2012). These observations suggest that METH can induce differential changes in the acetylation patterns of histones. Cadet, in collaboration with Bisagno, demonstrated that METH can alter the deacetylation of histones in brain regions involved in reward circuits by using a self-administration (SA) animal model (Cadet and Jayanthi, 2021). Further studies have shown that levels of HDAC2, HDAC8, and HDAC9 are elevated in the DS of METH-addicted mice but are reduced in the NAc. A particular study identified 14 SNPs near HDAC3 for genetic analysis to compare the genetic variations between METH-addicted patients and controls. The findings revealed that the SNP rs14251 was significantly associated with susceptibility to METH addiction, with the A allele increasing the risk (Xiao et al., 2022). These experimental results support the hypothesis that HDAC activity mediates the process of METH addiction. Moreover, the class I histone deacetylase inhibitor (HDACI), valproic acid (VPA), has been shown to attenuate behavioural sensitisation (Coccurello et al., 2007). The administration of neutralizing antibodies (nAbs) and VPA to the PFC, VTRs, AMG, and DC of mice has been shown to mitigate METH-induced behavioural sensitisation (Arent et al., 2011).It has been demonstrated that chronic injection of METH in mice results in specific binding of H4K16ac and reduced expression of GluA1 and GluA2 of the glutamate family (Jayanthi et al., 2014). Furthermore, a specific thiazolidinedione-based histone deacetylase 6 inhibitor (HDAC6I) has been shown to normalize the abundance of acetylated alpha microtubule proteins and reverse METH-induced morphological changes in neuroblastoma cell lines (Sharma et al., 2019). Another HDAC6I, MeBib, derived from a benzimidazole scaffold, has been employed in studies on mice, indicating that MeBib effectively reduces METH craving. Researchers have also genetically modified mice to lack the HDAC5 gene, finding that this alteration reduces the mice’s seeking behavior for METH (Kim et al., 2020). Moreover, HDAC5 knockout mice exhibited increased expression of target genes of HDAC1, HDAC4, and HDAC5, specifically Gnb4 and Suv39h1 (Li et al., 2018; Gonzalez et al., 2020). During acute METH injections, increased histone H3 acetylation levels have been demonstrated, along with enhanced expression of several synaptic plasticity genes (Shibasaki et al., 2011).Furthermore, some researchers have demonstrated that increased METH craving in mice is associated with the upregulation of HATs, specifically such as KAT4 and KAT5, in brain signaling pathways (Cadet et al., 2019). The frequency of active lever responses, indicative of METH SA, was diminished in rats pre-treated with HDAC6 inhibitors. METH exposure upregulated the expression of GluN2B-containing NMDA receptor subunits and triggered the concurrent activation of the extracellular signal-regulated kinase (ERK)/cAMP response element-binding protein (CREB)/brain-derived neurotrophic factor (BDNF) pathways in the hippocampus (Kim et al., 2020). These METH-induced neural circuitry changes were mitigated by HDAC6 inhibitors, suggesting their potential to attenuate METH addiction behaviors in rats. Based on the current evidence from both practical and theoretical studies, reducing drug craving in addicted groups through HDAC inhibitors targeting specific HDACs represents a highly promising approach in the quest for METH cessation (Werner et al., 2021). Moreover, research has demonstrated that the administration of high concentrations of HDACI resulted in increased acetylation of histone H1, primarily due to the suppression of HDAC3 activity. Low concentrations of these inhibitors preferentially increased α-tubulin acetylation, implicating the selective inhibition of HDAC6. Research conducted by Chiranjeev et al., in 2019 suggests that the biochemical mechanism underlying methamphetamine-induced morphological alterations in human neuroblastoma cells is linked to disrupted acetylation of α-tubulin. In the human neuroblastoma SH-SY5Y cell line, the HDAC6 inhibitor was observed to induce an increase in the acetylation level of α-tubulin by inhibiting HDAC6 in a dose-dependent manner. Furthermore, the HDAC6 inhibitor was demonstrated to effectively reverse methamphetamine-induced morphological changes in SH-SY5Y cells by regulating the acetylation level of α-tubulin. These epigenetic modulations were found to counteract the morphological alterations induced by METH in human neuroblastoma SH-SY5Y cells (Sharma et al., 2019). Indeed, there have been clinical examples of using HDACIs for cancer treatment. If we can successfully study the mechanism by which HDAC expression mediates the behavior of METH addiction in animal models, then clinical HDAC inhibitors might also be investigated as a potential treatment for METH addiction.

A clear insight from the available studies is that histone acetylation plays a key regulatory role in the mechanism of addiction to METH. This regulation involves modification of histone acetylation levels, as well as changes in the expression of the closely related HDACs, HATs, histone methyltransferases, and histone lysine demethylases. Of particular interest is the observation that neurotransmitters, receptors, and signaling pathways were significantly altered by histone acetylation following METH administration. The study further revealed that these modifications exhibited significant variability in specific brain regions strongly associated with METH addiction, such as the PFC, Hip, DS, and NAc. This variability may be attributed to a multitude of factors, including the timing of METH administration, dosage, self-administration procedures, conditioned place preference (CPP), as well as different brain regions and behavioral patterns (e.g., seeking, craving, relapsing, CPP expression, consolidation, fading, recovery, and motor sensitization). Consequently, histone acetylation at distinct locations may assume disparate roles in diverse neuronal subtypes (e.g., DA, 5-HT, Glu neurons), offering novel insights into the intricate mechanisms underlying METH addiction.

### 4.2 Methamphetamine addiction and histone methylation

Histone methylation is a covalent modification that occurs primarily on basic residues such as lysine 4, 9, 27, 36 and arginine 2, 8, 17, 26. Its role in the regulation of gene expression is more complex than that of acetylation. Methylation at different sites and the degree of methylation can trigger diverse effects, including both activating and repressing transcription (Penard-Lacronique and Bernard, 2016). Histone methylation marks are often associated with the activation, extension, or repression of gene expression. The principal methylations associated with gene activation are H3K4, H3K36, and H3K79. In contrast, H3K9me3 (trimethylation of histone H3 lysine 9) and H3K27me3 (trimethylation of lysine at position 27 of histone H3) are typically concentrated in heterochromatin regions and is associated with gene silencing. H4K20 methylation can serve as an indicator of activation. The extent of methylation determines whether the outcome is repression or activation. H4K20me1 (monomethylation of histone H4 lysine 20) is commonly associated with gene activation, whereas H4K20me3 (trimethylation of histone H4 lysine 20) is associated with gene silencing and heterochromatin formation. In general, histone methylation tends to result in gene silencing, whereas demethylation is typically associated with gene activation.

It has been demonstrated that the mRNA expression levels of the chemokine receptor CCR2 in the nucleus accumbens (NAc) of behaviorally sensitised mice following intermittent repeated injections of METH were increased. This increase was associated with enhanced trimethylation of histone H3 at lysine 4 (H3K4me3) at the CCR2 promoter in the NAc (Jayanthi et al., 2021; Ikegami et al., 2010). Additionally, METH-related memory in the NAc was accompanied by an increase in H3K4me2/3. These changes were secondary to enhanced expression of histone-lysine N-methyltransferase 2A (KMT2A), also known as myeloid/lymphoid or mixed-lineage leukaemia 1 (MLL1) (Greer and Shi, 2012). The researchers demonstrated that KMT2A and MLL1 can disrupt addictive behavioral memory by decreasing KMT2A and H3K4me3 abundance, as well as decreasing CPP for METH using small interfering RNA (siRNA) delivery (Aguilar-Valles et al., 2014). It was demonstrated that METH-associated memory is accompanied by an increase in H3K4me2/3 in the NAc, and that these changes are due to enhanced expression of KMT2A. The reduction of KMT2A and H3K4me3 levels by siRNA has been observed to diminish CPP in METH, thereby suggesting the potential for the development of novel therapeutic strategies for METH addiction. Although studies have confirmed that KMT2A and MLL1 play a role in addiction memory by influencing gene expression through the regulation of H3K4me2/3 levels during METH addiction, However, the broad functions and multiple targets of KMT2A/MLL1 suggest that its role may extend beyond addiction memory. The specificity of this regulatory mechanism remains to be verified. Further exploration of the interactions between KMT2A/MLL1 and other epigenetic modifications may facilitate the construction of a multilevel gene regulatory network. This will contribute to a comprehensive understanding of the complex regulatory mechanisms of epigenetics in addiction and identify new therapeutic targets. The use of siRNAs to reduce the abundance of KMT2A and H3K4me3 to interfere with addictive behaviours demonstrates potential therapeutic avenues. However, there are several challenges to be addressed before these technologies can be translated into clinical practice. These include the efficiency and specificity of siRNA delivery *in vivo*, the long-term effects, and the possible side effects. Further research is needed to assess the feasibility and effectiveness of these technologies in humans.

### 4.3 Methamphetamine addiction and histone ubiquitination

It is becoming increasingly clear that histone acetylation, methylation, and phosphorylation are all correlated with drug addiction. However, studies on histone ubiquitination during drug addiction are less frequently reported. It is now known that the process of METH addiction is accompanied by an increase in dopamine expression. This leads to an elevation in the level of reactive oxygen species (ROS) in neurons, which subsequently results in dysfunction of the ubiquitin-proteasome system (Martin et al., 2012). Related studies have shown that dopamine inhibits histone H2B ubiquitination. Upon application of dopamine to SH-SY5Y cells, RNF20, the primary enzyme responsible for histone H2B ubiquitination, is reduced in activity, thereby decreasing the expression of H2Bub1 (Martin et al., 2012). The microscopic process is as follows: during the development of METH-induced behavioral sensitization, METH molecules enter the transmitter vesicles through the presynaptic membrane, leading to the release of large amounts of monoamine neurotransmitters from the vesicles into the synaptic cleft. These neurotransmitters then block the presynaptic membrane’s ability to reuptake monoamine neurotransmitters, leading to the accumulation of a large amount of monoamine neurotransmitters in the synaptic cleft (Zahniser and Sorkin, 2009). These actions increase the secretion and action levels of monoamine neurotransmitters in the synaptic cleft. Monoamine transmitters, particularly dopamine, are secreted and acted upon more frequently in individuals exhibiting these behaviors. This results in deviations in histone ubiquitination levels, a stronger cyclic stimulation of the reward mechanism, and an increase in the organism’s craving for METH.

A ubiquitin protein ligase, designated Parkin, has been a subject of considerable interest in recent studies (Sharma et al., 2021). This enzyme exhibits neuroprotective and anti-inflammatory properties and is closely related to several biological processes, including dopaminergic and glutamatergic neurotransmission processes, energy metabolism, cytoskeletal arrangement, and oxidative stress and inflammation. Genetically engineered rat models have demonstrated that Parkin plays a pivotal role in regulating methamphetamine addictive behavior (Sharma et al., 2021). Rats lacking Parkin exhibited a greater tendency to become dependent on METH and remained in the METH environment for a longer duration than wild-type rats. In contrast, rats overexpressing Parkin displayed reduced METH uptake. Exposure to METH was shown to increase α-syn expression and polyubiquitination, while simultaneously decreasing Parkin levels and its interaction with α-syn (Meng et al., 2020). Notably, overexpression of Parkin was found to mitigate α-syn overexpression and neurotoxicity (Meng et al., 2020). This indicates that Parkin may reduce the neurotoxic effects of METH by regulating the degradation of α-syn. Furthermore, Parkin exerts an indirect influence on dopamine receptor function by affecting mitochondrial function and protein degradation pathways (Wang et al., 2018). This, in turn, regulates vesicular recycling and neurotransmitter release in dopamine neurons, ultimately affecting dopamine receptor signaling.

Given the limited efficacy of drug therapy in treating methamphetamine addiction, a class of ubiquitin-protein ligases functionally similar to Parkin represents a promising novel potential drug target for the treatment of methamphetamine addiction. It is anticipated that pharmacological enhancement of the expression of these enzymes *in vivo* will lead to a reduction in adaptive changes in neural pathways, thereby mitigating the effects of addiction.

## 5 Methamphetamine addiction and non-coding RNAs

Non-coding RNA (ncRNA) is RNA that does not code for proteins in the human genome (Lander et al., 2001). ncRNA can be broadly categorized into two types: housekeeping ncRNA and regulatory ncRNA. Housekeeping ncRNA is widely expressed and functions in maintaining basic cellular activities. On the other hand, regulatory ncRNAs play a crucial role in epigenetic mechanisms, essential for gene regulation at both transcriptional and post-transcriptional levels. Regulatory ncRNAs are typically classified based on their size, and current research focuses on microRNAs (miRNAs), long non-coding RNAs (lncRNAs), and circular RNAs (circRNAs), all of which play vital roles in regulatory processes (Kang et al., 2022). The following section will therefore examine the potential relationship between these three regulatory ncRNAs and METH neurotoxicity.

### 5.1 miRNAs and methamphetamine addiction

As non-coding RNAs, miRNAs are not involved in the translation of genes into proteins (Zhang et al., 2019). However, they play an important role in the processes preceding and following transcription, including those in the processes of neuronal plasticity and drug addiction (Schratt, 2009). Siegel et al. found that the expression of miR-29b and miR-138 in the NAc is associated with neuronal plasticity (Siegel et al., 2009). Chandrasekar and Dreyer (2011) demonstrated that miR-124 in NAc regulates cocaine-induced conditioned positional preference. Moreover, it has been demonstrated that miRNAs play a regulatory role in methamphetamine-mediated changes in neural dendritic spines and synaptic transmission (Wang et al., 2022). This reflects the pervasive nature of miRNAs in regulating drug addiction.

A genome-wide transcriptional analysis was conducted in the NAc and PFC, revealing that elevated levels of METH addiction-related molecules were accompanied by increased expression levels of several miRNAs (Cates et al., 2018). Furthermore, the levels of miRNAs 237, 296, and 501 were elevated in NAc when METH-induced conditioned position preference was observed (Yang et al., 2020). miRNAs in NAc: regulate genes involved in Wnt signalling and axon guidance. The Wnt/β-catenin pathway, an important pathway that transmits Wnt signals in cells, is involved in a variety of processes, including cell fate determination, development, and carcinogenesis. In neurons, this pathway is also involved in neurogenesis and synaptic plasticity. METH activates the Wnt/β-catenin pathway in NAc to promote dopamine release and sensitisation behaviour (Gu et al., 2020). Certain miRNAs associated with METH addiction, such as miR-124, miR-181a, and miR-9, can inhibit the activation of the Wnt/β-catenin pathway by targeting β-catenin or its downstream molecules, thus inhibiting METH-induced dopamine release and sensitisation behaviour. It was demonstrated that miR-181a-5p expression was reduced in rats that had been administered methamphetamine and subsequently developed an addiction. miR-181a can cause GABAAα1 ubiquitination in the PI3K/Akt pathway through the regulation of ERAD, which leads to a reduction in GABAAα1 expression in the dorsal striatum. This reduction in GABAAα1 expression in methamphetamine-addicted rats is associated with the induction of METH addiction (Wang et al., 2021). In METH-induced addicted rats, miRNA levels are dysregulated in the dorsal striatum as a consequence of METH intake. Specifically, METH-induced behavioural sensitisation in mice was found to be negatively correlated with the degree of development of the behavioural sensitisation.The measurement of Ago2-dependent miRNAs in NAc neurons revealed that miR-3068-5p disrupts METH-induced locomotor sensitization (Liu et al., 2021). These effects of Ago2/miR-3068-5p occur through interactions with the glutamate receptor, GluN1/Grin1 (Liu et al., 2021). Furthermore, Li et al. (2021) found that during METH-induced locomotor sensitization, increased expression levels of miR-128 were observed. AAV-mediated overexpression of miR-128 was found to significantly contribute to the development of addictive behaviors. Conversely, AAV-mediated inhibition of miR-128 expression was found to attenuate METH-induced addictive behavior.

A recent study has indicated that methamphetamine (METH) exposure upregulates α-synuclein (α-syn) in neurons, which may directly induce mitochondrial damage, myelin destruction, and synaptic failure. Furthermore, the accumulation of α-syn may also indirectly promote tau phosphorylation via tau kinase, glycogen synthase kinase 3β (GSK3β), and cell cycle protein-dependent kinase 5 (CDK5), leading to microtubule depolymerization and ultimately impaired fusion of autophagosomes and lysosomes (Ding et al., 2020). One of the key strategies to inhibit α-syn generation is to regulate the post-transcriptional level of α-syn by miRNAs. For instance, miRNA-7 (miR-7) is capable of targeting SNCA, the gene encoding α-synuclein (α-syn), in order to reduce α-syn expression and, as a consequence, to alleviate the neurological damage associated with Parkinson’s Disease (PD) (Hallacli et al., 2022). Furthermore, miR-7 expression was found to be reduced in basal state A53T α-synuclein overexpressing mice and elevated in α-synuclein knockout mice (Hallacli et al., 2022). This suggests that α-syn expression is not only negatively regulated by miR-7, but may also directly inhibit miR-7 expression.

In conclusion, it can be postulated that the administration of miRNA mimics or inhibitors may result in the modulation of specific miRNAs, thereby improving the behavioural manifestations of METH-induced dopamine release, sensitisation, relapse, and cognitive impairment (Zhao et al., 2022). Furthermore, some studies have demonstrated that the expression or function of miRNAs can be stably regulated by transducing vectors or expression systems containing specific miRNAs, thereby achieving a long-lasting therapeutic effect. Nevertheless, the selection of suitable target miRNAs and the instability of miRNAs, which necessitates their protection and delivery via appropriate vectors or modifications, remain areas for further exploration and research.

### 5.2 lncRNAs and methamphetamine addiction

Long non-coding RNAs (lncRNAs) are a class of non-coding RNAs (ncRNAs) over 200 nucleotides in length. They represent the largest class of ncRNAs currently annotated and identified in the human genome (Derrien et al., 2012). lncRNAs affect a multitude of important physiological processes, assuming a variety of roles through the recruitment of transcription factors or the inhibition of miRNA binding to mRNAs via sponging (Xu et al., 2023). Consequently, they participate in the regulation of target genes, such as chromatin modifiers, X-chromosome inactivators, enhancers, transcriptional regulators, and post-transcriptional regulators. As epigenetic factors, lncRNAs have been found to play a significant role in METH-induced behavioural sensitivity (Rezayof et al., 2023). Researchers have identified that METH induces alterations in lncRNA expression levels in mouse NAc through high-throughput sequencing. This approach has revealed that METH regulates five lncRNAs (Kcnq1ot1, Zfhx2as, Neat1, Neat2, and Miat) and corresponding genes that code for proteins (Zhu et al., 2015). These regulated genes are involved in synaptic transmission, which indirectly reflects the transmission pathways of related factors in the process of METH addiction. Ip et al. demonstrated that lncRNA Gomafu (also known as Miat) may be responsible for METH addiction by constructing a mouse knockout (KO) model of the Gomafu gene (Ip et al., 2016). This model exhibited an enhanced response to METH when the Miat was knocked out, which was associated with an increase in the release of dopamine in the nucleus accumbens. These findings confirm that the Miat may alter mouse behaviour by regulating gene expression or selectively splicing target genes. Zhu et al. (2016) demonstrated that METH administration resulted in alterations in lncRNA expression in the NAc of sensitized mice, with a predominant downregulation. These changes were hypothesized to be involved in METH-induced behavioural sensitization and addiction.

Long non-coding RNA MEG3 is a type of non-coding RNA that, while not encoding proteins, plays a crucial role in gene regulation (Li et al., 2022). PI3K (Phosphatidylinositol 3-Kinase) is involved in a process that activates Akt (protein kinase B), a key signaling molecule regulating cell growth, proliferation, survival, and metabolism. Akt is activated by PI3K, which subsequently triggers a series of intracellular responses promoting cell survival and growth. When MEG3 is upregulated, it may directly bind to PI3K or Akt proteins, thereby impeding their activation or function (Xu et al., 2023). For instance, MEG3 may inhibit PI3K activity by binding to the catalytic subunit p110 or the regulatory subunit p85, thereby reducing the production of PIP3. MEG3 may also exert an indirect inhibitory effect on the PI3K/Akt signaling pathway by regulating upstream regulators of this pathway, such as receptor tyrosine kinases (RTKs) and G protein-coupled receptors (GPCRs) (Xu et al., 2023). Furthermore, MEG3 has the ability to hinder the PI3K/Akt signaling cascade by modulating downstream effector molecules, including PTEN (phosphatase and tensin homologue), a significant tumor suppressor that inhibits the PI3K/Akt signaling cascade through dephosphorylating PIP3^[81^^,^^86]^. MEG3 may influence the expression of genes associated with the PI3K/Akt signaling pathway by modulating DNA methylation and histone modifications. Specifically, MEG3 may affect the activity of the PI3K/Akt signaling pathway by interacting with EZH2 (histone methyltransferase), which controls the methylation status of histone H3. MEG3 exerts its inhibitory effect on the PI3K/Akt signaling pathway through various mechanisms, and the reduction in PI3K/Akt pathway activity leads to decreased signals for neuronal overgrowth and survival, subsequently mitigating oxidative stress and inflammation, and protecting neurons (He et al., 2021). While there is a paucity of related studies, this series of regulatory changes offers a novel avenue for research into the mechanism of long non-coding RNA (lncRNA) regulation of METH addiction.

### 5.3 circRNAs and methamphetamine addiction

Circular RNAs (circRNAs) form a closed-loop structure with covalent bonds, lacking a polyadenylation tail at the 3′end and a cap structure at the 5′end (Jeck and Sharpless, 2014). This special structure protects circRNAs from nucleic acid exonuclease degradation and allows them to have a longer half-life than their parental mRNAs (Jeck and Sharpless, 2014). circRNAs can directly regulate transcription by interacting with mRNAs or long non-coding RNAs (lncRNAs), sponging of miRNAs, or binding to RNA-binding proteins (RBPs) (Memczak et al., 2013; Hansen et al., 2013). Previous studies have indicated that Cdr1as and Hipk3 may be associated with METH-induced behavioural sensitisation (Nan et al., 2017). In the recent study, the experimenters first cultured primary cortical neurons pretreated with METH and then subjected them to high-throughput RNA sequencing to screen for circRNA-specific expression. Subsequently, bioinformatics analysis was employed to predict the potential functions of the differentially expressed circRNAs, resulting in the identification of 119 circRNAs with upregulated expression and 44 with downregulated expression (Li et al., 2019). CircHomer1 was subsequently validated as a potential candidate for further investigation. METH addiction was assessed in mice using the CPP assay, and it was demonstrated that knockdown of circHomer1 could alleviate METH addiction by suppressing the expression of Bbc3, thereby attenuating METH-induced nerve damage (Li et al., 2019). At present, the majority of circRNA studies are focused on cancer and metabolism, with fewer investigations pertaining to circRNA and METH addiction. However, METH addiction is intricately linked to the metabolic processes of biological organisms. A small number of circRNAs can inhibit a multitude of miRNAs, and given the pivotal role that miRNAs play in METH addiction, it can be postulated that circRNAs are also intricately linked to METH addiction (Table 1).

**TABLE 1:** Table of changes at the level of biomolecules under epigenetic action.

Epigenetic mechanisms	Brain sites of action	Molecular changes	Target points
DNA methylation	Hippocampus, Dorsal striatum, Prefrontal cortex, Medial prefrontal cortex, Nucleus accumbens	KDM5C↑, KMT2A↑, LINE↑, Shati/NAT8L↑, MLL1↑, HMT↑, DNMT↑↓(In particular, DNMT3A, DNMT3B, etc.) MeCP↑↓ HADC2↑↓	BDNF↑↓, OT↓, K+ channel↑↓, Syp↑↓, a-Syn↑, Glu↑, GluA1↑ GluA2↑ LINE-1↑, GABA↓ CHN2↑ PVALB↑
Histone acetylation	Hippocampus, Dorsal striatum, Prefrontal cortex, Nucleus accumbens	HDACs↑↓(Including 1, 2, 3, 4, etc.) HATs↑(In particular, KAT4, KAT5, etc.) H3↑↓, H4↑↓, H2BAc↑, H3K9Ac↑↓, H3K18Ac↑↓ H4K5Ac↑↓ H4K8Ac↑↓ H4K12Ac↑↓	DA1↑, DA2↑, HCRTR1↓↑ HCRTR2↑, HRH1, 3↑, NMDA↑, GluA1↑↓ GluA2↑↓
Histone methylation	Hippocampus, Dorsal striatum, Prefrontal cortex, Medial prefrontal cortex, Nucleus accumbens	KDM5C↑, KMT2A↑, H3K4↑, LINE↑, Shati/NAT8L↑, HMT↑ DNMT↑↓ MeCP↑↓	BDNF↑, OT↓, K+ channel↑↓, Syp↑↓, a-Syn↑,Glu↑, GluA1↑, GluA2↑ LINE-1↑, GABA↓, BDNF↑
Histone ubiquitination	Basolateral amygdala, Central amygdala	Parkin↑, α-syn↑, SYVN1↓, RNF20↓	DA1↑,DA2↑, NMDA↑, AMPA↑, GABAAα1↑, H2Bub1↓
miRNAs	Hippocampus, Dorsal striatum, Nucleus accumbens, Ventral tegmental area of midbrain	miR-128↑, 237↑, 296↑, 501↑31-3p↑,34a-5p↑, 183-5p↑, 9a-5p↑, 369-3p↑, 29a↑, 181a/d↑	PKG↑, PI3K↑, Wnt↑, Ago2↑, NRG-1↑, BDNF↑, GluN1↑
lncRNAs	Hippocampus, Nucleus accumbens	Kcnqlot1↑, Zfhx2as↑, Neat1↑, Neat2↑, Miat↑	Camk4↑ CREB1↑ AMPA α1↑, CREB-binding protein↑
circRNAs	Hippocampus, Prefrontal cortex	Cdrlas↑Hipk3↑	Bbc3↓

## 6 Conclusion and future directions

The process of METH-induced neurotoxicity involves numerous changes in molecular mechanisms, with alterations to many epigenetic factors playing a key role in enhancing the craving for METH through adaptive changes in the reward mechanism. For instance, modulations like DNA methylation, histone modifications, and non-coding RNAs have the potential to be effective targets for mitigating METH neurotoxicity and deserve further exploration. Furthermore, numerous research experiments on methamphetamine addiction have identified and confirmed the dynamic response changes of numerous brain regions and gene loci throughout the addiction process. These reflect the target sites of methamphetamine action within the organism. The inhibitory reverse regulation of these brain regions and gene loci following drug exposure may potentially restore them to their normal level, thereby offering a promising avenue for methamphetamine treatment. However, as far as the current study is concerned, it still has more limitations. Firstly, the relationship between various types of factors in epigenetic mechanisms and METH-induced behavioural sensitisation has not been fully explored. Secondly, the researchers mostly used animal models to investigate the indexes related to METH-induced behavioural sensitisation, despite the difference in mechanism between animal organisms and human bodies. The mode of drug administration in human beings is autonomous, but the animal model is passive, and it remains to be investigated whether the mode of drug administration will lead to changes in the experimental data. In the case of the most commonly used rat model for experiments, it can be observed that more than 70% of the DARs associated with methamphetamine (METH) exposure that are conserved between rats and humans can only indicate that these regions have maintained somewhat similar gene regulatory functions during evolution. As previously stated in the article, an additional 10% of the DARs associated with methamphetamine (METH) exposure are rat-specific. The rat model possesses distinctive species-specific characteristics in the field of addiction research, which introduces a degree of uncertainty when extrapolating data from animal models to the human organism. Moreover, the majority of experiments only studied a single aspect of the epigenetic mechanism, and the experimental design was not comprehensive. This has led to the conclusion that gene expression is co-regulated by multiple epigenetic factors, which is a limited interpretation. With the development of high-throughput technology and bioinformatics analysis tools, we are expected to reveal more about the regulatory roles between epigenetics and transcription. In the future, researchers should further optimise the study of the full range of epigenetic mechanisms of methamphetamine neurotoxicity, with the aim of providing more practical data and a more rigorous theoretical basis for finding innovative targets for methamphetamine cessation.

## References

[B1] Aguilar-Valles A. Vaissiere T. Griggs E. M. Mikaelsson M. A. Takacs I. F. Young E. J. (2014). Methamphetamine-associated memory is regulated by a writer and an eraser of permissive histone methylation. Biol. Psychiatry 76 (1), 57–65. 10.1016/j.biopsych.2013.09.014 24183790 PMC4024089

[B2] Arent C. O. Valvassori S. S. Fries G. R. Stertz L. Ferreira C. L. Lopes-Borges J. (2011). Neuroanatomical profile of antimaniac effects of histone deacetylases inhibitors. Mol. Neurobiol. 43 (3), 207–214. 10.1007/s12035-011-8178-0 21424678

[B3] Baxter E. Windloch K. Gannon F. Lee J. S. (2014). Epigenetic regulation in cancer progression. Cell Biosci. 4, 45. 10.1186/2045-3701-4-45 25949794 PMC4422217

[B4] Bhat K. P. Umit K. H. Jin J. Gozani O. (2021). Epigenetics and beyond: targeting writers of protein lysine methylation to treat disease. Nat. Rev. Drug Discov. 20 (4), 265–286. 10.1038/s41573-020-00108-x 33469207 PMC8035164

[B5] Cadet J. L. Brannock C. Krasnova I. N. Jayanthi S. Ladenheim B. Mccoy M. T. (2017). Genome-wide DNA hydroxymethylation identifies potassium channels in the nucleus accumbens as discriminators of methamphetamine addiction and abstinence. Mol. Psychiatry 22 (8), 1196–1204. 10.1038/mp.2016.48 27046646 PMC7405865

[B6] Cadet J. L. Jayanthi S. (2021). Epigenetics of addiction. Neurochem. Int. 147, 105069. 10.1016/j.neuint.2021.105069 33992741 PMC8260024

[B7] Cadet J. L. Patel R. Jayanthi S. (2019). Compulsive methamphetamine taking and abstinence in the presence of adverse consequences: epigenetic and transcriptional consequences in the rat brain. Pharmacol. Biochem. Behav. 179, 98–108. 10.1016/j.pbb.2019.02.009 30797763

[B8] Cates H. M. Li X. Purushothaman I. Kennedy P. J. Shen L. Shaham Y. (2018). Genome-wide transcriptional profiling of central amygdala and orbitofrontal cortex during incubation of methamphetamine craving. Neuropsychopharmacology 43 (12), 2426–2434. 10.1038/s41386-018-0158-x 30072726 PMC6180053

[B9] Chandrasekar V. Dreyer J. L. (2011). Regulation of MiR-124, Let-7d, and MiR-181a in the accumbens affects the expression, extinction, and reinstatement of cocaine-induced conditioned place preference. Neuropsychopharmacology 36 (6), 1149–1164. 10.1038/npp.2010.250 21307844 PMC3079833

[B10] Coccurello R. Caprioli A. Ghirardi O. Virmani A. (2007). Valproate and acetyl-L-carnitine prevent methamphetamine-induced behavioral sensitization in mice. Ann. N. Y. Acad. Sci. 1122, 260–275. 10.1196/annals.1403.019 18077579

[B11] Compere S. J. Palmiter R. D. (1981). DNA methylation controls the inducibility of the mouse metallothionein-I gene lymphoid cells. Cell 25 (1), 233–240. 10.1016/0092-8674(81)90248-8 6168387

[B12] Derrien T. Johnson R. Bussotti G. Tanzer A. Djebali S. Tilgner H. (2012). The GENCODE v7 catalog of human long noncoding RNAs: analysis of their gene structure, evolution, and expression. Genome Res. 22 (9), 1775–1789. 10.1101/gr.132159.111 22955988 PMC3431493

[B13] Ding J. Hu S. Meng Y. Li C. Huang J. He Y. (2020). Alpha-Synuclein deficiency ameliorates chronic methamphetamine induced neurodegeneration in mice. Toxicology 438, 152461. 10.1016/j.tox.2020.152461 32278788

[B14] Eyerman D. J. Yamamoto B. K. (2007). A rapid oxidation and persistent decrease in the vesicular monoamine transporter 2 after methamphetamine. J. Neurochem. 103 (3), 1219–1227. 10.1111/j.1471-4159.2007.04837.x 17683483

[B15] Fan X. Y. Yang J. Y. Dong Y. X. Hou Y. Liu S. Wu C. F. (2020). Oxytocin inhibits methamphetamine-associated learning and memory alterations by regulating DNA methylation at the Synaptophysin promoter. Addict. Biol. 25 (1), e12697. 10.1111/adb.12697 30585381

[B16] Feil R. Fraga M. F. (2012). Epigenetics and the environment: emerging patterns and implications. Nat. Rev. Genet. 13 (2), 97–109. 10.1038/nrg3142 22215131

[B17] Fleckenstein A. E. Volz T. J. Riddle E. L. Gibb J. W. Hanson G. R. (2007). New insights into the mechanism of action of amphetamines. Annu. Rev. Pharmacol. Toxicol. 47, 681–698. 10.1146/annurev.pharmtox.47.120505.105140 17209801

[B18] Godino A. Jayanthi S. Cadet J. L. (2015). Epigenetic landscape of amphetamine and methamphetamine addiction in rodents. Epigenetics 10 (7), 574–580. 10.1080/15592294.2015.1055441 26023847 PMC4622560

[B19] Gonzalez B. Bernardi A. Torres O. V. Jayanthi S. Gomez N. Sosa M. H. (2020). HDAC superfamily promoters acetylation is differentially regulated by modafinil and methamphetamine in the mouse medial prefrontal cortex. Addict. Biol. 25 (2), e12737. 10.1111/adb.12737 30811820 PMC8388191

[B20] Gonzalez B. Torres O. V. Jayanthi S. Gomez N. Sosa M. H. Bernardi A. (2019). The effects of single-dose injections of modafinil and methamphetamine on epigenetic and functional markers in the mouse medial prefrontal cortex: potential role of dopamine receptors. Prog. Neuropsychopharmacol. Biol. Psychiatry 88, 222–234. 10.1016/j.pnpbp.2018.07.019 30056065 PMC8424782

[B21] Greer E. L. Shi Y. (2012). Histone methylation: a dynamic mark in health, disease and inheritance. Nat. Rev. Genet. 13 (5), 343–357. 10.1038/nrg3173 22473383 PMC4073795

[B22] Gu W. J. Zhang C. Zhong Y. Luo J. Zhang C. Y. Zhang C. (2020). Altered serum microRNA expression profile in subjects with heroin and methamphetamine use disorder. Biomed. Pharmacother. 125, 109918. 10.1016/j.biopha.2020.109918 32036213

[B23] Hallacli E. Kayatekin C. Nazeen S. Wang X. H. Sheinkopf Z. Sathyakumar S. (2022). The Parkinson's disease protein alpha-synuclein is a modulator of processing bodies and mRNA stability. Cell 185 (12), 2035–2056.e33. 10.1016/j.cell.2022.05.008 35688132 PMC9394447

[B24] Han B. Compton W. M. Jones C. M. Einstein E. B. Volkow N. D. (2021). Methamphetamine Use, methamphetamine use disorder, and associated overdose deaths among US adults. JAMA Psychiatry 78 (12), 1329–1342. 10.1001/jamapsychiatry.2021.2588 34550301 PMC8459304

[B25] Hansen T. B. Jensen T. I. Clausen B. H. Bramsen J. B. Finsen B. Damgaard C. K. (2013). Natural RNA circles function as efficient microRNA sponges. Nature 495 (7441), 384–388. 10.1038/nature11993 23446346

[B26] Hao L. Luo T. Dong H. Tang A. Hao W. (2017). CHN2 promoter methylation change may Be associated with methamphetamine dependence. Shanghai Arch. Psychiatry 29 (6), 357–364. 10.11919/j.issn.1002-0829.217100 29719347 PMC5925587

[B27] He Y. Sun M. M. Zhang G. G. Yang J. Chen K. S. Xu W. W. (2021). Targeting PI3K/Akt signal transduction for cancer therapy. Signal Transduct. Target Ther. 6 (1), 425. 10.1038/s41392-021-00828-5 34916492 PMC8677728

[B28] Hong S. J. Shen B. Y. Sun R. J. Yang G. M. Duan C. M. Nie Q. Y. (2021). Current situation of methamphetamine abuse and related research progress. Fa Yi Xue Za Zhi 37 (6), 763–775. 10.12116/j.issn.1004-5619.2021.310202 35243841

[B29] Ikegami D. Narita M. Imai S. Miyashita K. Tamura R. Narita M. (2010). Epigenetic modulation at the CCR2 gene correlates with the maintenance of behavioral sensitization to methamphetamine. Addict. Biol. 15 (3), 358–361. 10.1111/j.1369-1600.2010.00219.x 20624155

[B30] Ip J. Y. Sone M. Nashiki C. Pan Q. Kitaichi K. Yanaka K. (2016). Gomafu lncRNA knockout mice exhibit mild hyperactivity with enhanced responsiveness to the psychostimulant methamphetamine. Sci. Rep. 6, 27204. 10.1038/srep27204 27251103 PMC4890022

[B31] Jayanthi S. Gonzalez B. Mccoy M. T. Ladenheim B. Bisagno V. Cadet J. L. (2018). Methamphetamine induces TET1-and TET3-dependent DNA hydroxymethylation of Crh and Avp genes in the rat nucleus accumbens. Mol. Neurobiol. 55 (6), 5154–5166. 10.1007/s12035-017-0750-9 28842817 PMC5948251

[B32] Jayanthi S. Mccoy M. T. Cadet J. L. (2021). Epigenetic regulatory dynamics in models of methamphetamine-use disorder. Genes (Basel) 12 (10), 1614. 10.3390/genes12101614 34681009 PMC8535492

[B33] Jayanthi S. Mccoy M. T. Chen B. Britt J. P. Kourrich S. Yau H. J. (2014). Methamphetamine downregulates striatal glutamate receptors via diverse epigenetic mechanisms. Biol. Psychiatry 76 (1), 47–56. 10.1016/j.biopsych.2013.09.034 24239129 PMC3989474

[B34] Jayanthi S. Torres O. V. Ladenheim B. Cadet J. L. (2020). A single prior injection of methamphetamine enhances methamphetamine self-administration (SA) and blocks SA-induced changes in DNA methylation and mRNA expression of potassium channels in the rat nucleus accumbens. Mol. Neurobiol. 57 (3), 1459–1472. 10.1007/s12035-019-01830-3 31758400 PMC7060962

[B35] Jeck W. R. Sharpless N. E. (2014). Detecting and characterizing circular RNAs. Nat. Biotechnol. 32 (5), 453–461. 10.1038/nbt.2890 24811520 PMC4121655

[B36] Jones P. A. (2012). Functions of DNA methylation: islands, start sites, gene bodies and beyond. Nat. Rev. Genet. 13 (7), 484–492. 10.1038/nrg3230 22641018

[B37] Kahlig K. M. Lute B. J. Wei Y. Loland C. J. Gether U. Javitch J. A. (2006). Regulation of dopamine transporter trafficking by intracellular amphetamine. Mol. Pharmacol. 70 (2), 542–548. 10.1124/mol.106.023952 16684900

[B38] Kang J. Y. Wen Z. Pan D. Zhang Y. Li Q. Zhong A. (2022). LLPS of FXR1 drives spermiogenesis by activating translation of stored mRNAs. Science 377 (6607), eabj6647. 10.1126/science.abj6647 35951695

[B39] Kim B. Jha S. Seo J. H. Jeong C. H. Lee S. Lee S. (2020). MeBib suppressed methamphetamine self-administration response via inhibition of BDNF/ERK/CREB signal pathway in the Hippocampus. Biomol. Ther. Seoul. 28 (6), 519–526. 10.4062/biomolther.2020.041 32466633 PMC7585641

[B40] Kirtana R. Manna S. Patra S. K. (2020). Molecular mechanisms of KDM5A in cellular functions: facets during development and disease. Exp. Cell Res. 396 (2), 112314. 10.1016/j.yexcr.2020.112314 33010254

[B41] Lammel S. Lim B. K. Malenka R. C. (2014). Reward and aversion in a heterogeneous midbrain dopamine system. Neuropharmacology 76 Pt B (0 0), 351–359. 10.1016/j.neuropharm.2013.03.019 23578393 PMC3778102

[B42] Lander E. S. Linton L. M. Birren B. Nusbaum C. Zody M. C. Baldwin J. (2001). Initial sequencing and analysis of the human genome. Nature 409 (6822), 860–921. 10.1038/35057062 11237011

[B43] Laplant Q. Vialou V. Covington H. R. Dumitriu D. Feng J. Warren B. L. (2010). Dnmt3a regulates emotional behavior and spine plasticity in the nucleus accumbens. Nat. Neurosci. 13 (9), 1137–1143. 10.1038/nn.2619 20729844 PMC2928863

[B44] Li E. Zhang Y. (2014). DNA methylation in mammals. Cold Spring Harb. Perspect. Biol. 6 (5), a019133. 10.1101/cshperspect.a019133 24789823 PMC3996472

[B45] Li J. Shi Q. Wang Q. Tan X. Pang K. Liu X. (2019). Profiling circular RNA in methamphetamine-treated primary cortical neurons identified novel circRNAs related to methamphetamine addiction. Neurosci. Lett. 701, 146–153. 10.1016/j.neulet.2019.02.032 30797870

[B46] Li J. Zhu L. Su H. Liu D. Yan Z. Ni T. (2021). Regulation of miR-128 in the nucleus accumbens affects methamphetamine-induced behavioral sensitization by modulating proteins involved in neuroplasticity. Addict. Biol. 26 (1), e12881. 10.1111/adb.12881 32058631

[B47] Li X. Carreria M. B. Witonsky K. R. Zeric T. Lofaro O. M. Bossert J. M. (2018). Role of dorsal striatum histone deacetylase 5 in incubation of methamphetamine craving. Biol. Psychiatry 84 (3), 213–222. 10.1016/j.biopsych.2017.12.008 29397902 PMC6026084

[B48] Li Z. Gao J. Sun D. Jiao Q. Ma J. Cui W. (2022). LncRNA MEG3: potential stock for precision treatment of cardiovascular diseases. Front. Pharmacol. 13, 1045501. 10.3389/fphar.2022.1045501 36523500 PMC9744949

[B49] Liang D. Yan R. Long X. Ji D. Song B. Wang M. (2024). Distinct dynamics of parental 5-hydroxymethylcytosine during human preimplantation development regulate early lineage gene expression. Nat. Cell Biol. 26 (9), 1458–1469. 10.1038/s41556-024-01475-y 39080410 PMC11392820

[B50] Liu D. Liang M. Zhu L. Zhou T. T. Wang Y. Wang R. (2021). Potential ago2/miR-3068-5p cascades in the nucleus accumbens contribute to methamphetamine-induced locomotor sensitization of mice. Front. Pharmacol. 12, 708034. 10.3389/fphar.2021.708034 34483916 PMC8414410

[B51] Martin T. A. Jayanthi S. Mccoy M. T. Brannock C. Ladenheim B. Garrett T. (2012). Methamphetamine causes differential alterations in gene expression and patterns of histone acetylation/hypoacetylation in the rat nucleus accumbens. PLoS One 7 (3), e34236. 10.1371/journal.pone.0034236 22470541 PMC3314616

[B52] Memczak S. Jens M. Elefsinioti A. Torti F. Krueger J. Rybak A. (2013). Circular RNAs are a large class of animal RNAs with regulatory potency. Nature 495 (7441), 333–338. 10.1038/nature11928 23446348

[B53] Meng Y. Qiao H. Ding J. He Y. Fan H. Li C. (2020). Effect of Parkin on methamphetamine‐induced α‐synuclein degradation dysfunction *in vitro* and *in vivo* . Brain Behav. 10 (4), e1574. 10.1002/brb3.1574 PMC7177580 32086884

[B54] Miao B. Xing X. Bazylianska V. Madden P. Moszczynska A. Zhang B. (2023). Methamphetamine-induced region-specific transcriptomic and epigenetic changes in the brain of male rats. Commun. Biol. 6 (1), 991. 10.1038/s42003-023-05355-3 37758941 PMC10533900

[B55] Moszczynska A. (2020). Differential responses of LINE-1 in the dentate gyrus, striatum and prefrontal cortex to chronic neurotoxic methamphetamine: a study in rat brain. Genes (Basel) 11 (4), 364. 10.3390/genes11040364 32231019 PMC7230251

[B56] Mottamal M. Zheng S. Huang T. L. Wang G. (2015). Histone deacetylase inhibitors in clinical studies as templates for new anticancer agents. Molecules 20 (3), 3898–3941. 10.3390/molecules20033898 25738536 PMC4372801

[B57] Nan A. Chen L. Zhang N. Liu Z. Yang T. Wang Z. (2017). A novel regulatory network among LncRpa, CircRar1, MiR-671 and apoptotic genes promotes lead-induced neuronal cell apoptosis. Arch. Toxicol. 91 (4), 1671–1684. 10.1007/s00204-016-1837-1 27604105 PMC5364257

[B58] Nativio R. Lan Y. Donahue G. Sidoli S. Berson A. Srinivasan A. R. (2020). An integrated multi-omics approach identifies epigenetic alterations associated with Alzheimer's disease. Nat. Genet. 52 (10), 1024–1035. 10.1038/s41588-020-0696-0 32989324 PMC8098004

[B59] Numachi Y. Shen H. Yoshida S. Fujiyama K. Toda S. Matsuoka H. (2007). Methamphetamine alters expression of DNA methyltransferase 1 mRNA in rat brain. Neurosci. Lett. 414 (3), 213–217. 10.1016/j.neulet.2006.12.052 17254711

[B60] Penard-Lacronique V. Bernard O. A. (2016). IDH1, histone methylation, and so forth. Cancer Cell 30 (2), 501–194. 10.1016/j.ccell.2016.08.010 27622339

[B61] Proudnikov D. Laforge K. S. Hofflich H. Levenstien M. Gordon D. Barral S. (2006). Association analysis of polymorphisms in serotonin 1B receptor (HTR1B) gene with heroin addiction: a comparison of molecular and statistically estimated haplotypes. Pharmacogenet Genomics 16 (1), 25–36. 10.1097/01.fpc.0000182782.87932.d6 16344719

[B62] Rezayof A. Ghasemzadeh Z. Sahafi O. H. (2023). Addictive drugs modify neurogenesis, synaptogenesis and synaptic plasticity to impair memory formation through neurotransmitter imbalances and signaling dysfunction. Neurochem. Int. 169, 105572. 10.1016/j.neuint.2023.105572 37423274

[B63] Ross S. Peselow E. (2009). The neurobiology of addictive disorders. Clin. Neuropharmacol. 32 (5), 269–276. 10.1097/wnf.0b013e3181a9163c 19834992

[B64] Scearce-Levie K. Viswanathan S. S. Hen R. (1999). Locomotor response to MDMA is attenuated in knockout mice lacking the 5-HT1B receptor. Psychopharmacol. Berl. 141 (2), 154–161. 10.1007/s002130050819 9952039

[B65] Schinder A. F. Olson E. C. Spitzer N. C. Montal M. (1996). Mitochondrial dysfunction is a primary event in glutamate neurotoxicity. J. Neurosci. 16 (19), 6125–6133. 10.1523/JNEUROSCI.16-19-06125.1996 8815895 PMC6579180

[B66] Schratt G. (2009). microRNAs at the synapse. Nat. Rev. Neurosci. 10 (12), 842–849. 10.1038/nrn2763 19888283

[B67] Sharma A. Harutyunyan A. Schneider B. L. Moszczynska A. (2021). Parkin regulates drug-taking behavior in rat model of methamphetamine use disorder. Transl. Psychiatry 11 (1), 293. 10.1038/s41398-021-01387-7 34001858 PMC8129108

[B68] Sharma C. Oh Y. J. Park B. Lee S. Jeong C. H. Lee S. (2019). Development of thiazolidinedione-based HDAC6 inhibitors to overcome methamphetamine addiction. Int. J. Mol. Sci. 20 (24), 6213. 10.3390/ijms20246213 31835389 PMC6940941

[B69] Shibasaki M. Mizuno K. Kurokawa K. Ohkuma S. (2011). L-type voltage-dependent calcium channels facilitate acetylation of histone H3 through PKCγ phosphorylation in mice with methamphetamine-induced place preference. J. Neurochem. 118 (6), 1056–1066. 10.1111/j.1471-4159.2011.07387.x 21781114

[B70] Shin E. J. Jeong J. H. Hwang Y. Sharma N. Dang D. K. Nguyen B. T. (2021). Methamphetamine-induced dopaminergic neurotoxicity as a model of Parkinson's disease. Arch. Pharm. Res. 44 (7), 668–688. 10.1007/s12272-021-01341-7 34286473

[B71] Siegel G. Obernosterer G. Fiore R. Oehmen M. Bicker S. Christensen M. (2009). A functional screen implicates microRNA-138-dependent regulation of the depalmitoylation enzyme APT1 in dendritic spine morphogenesis. Nat. Cell Biol. 11 (6), 705–716. 10.1038/ncb1876 19465924 PMC3595613

[B72] Thomas D. M. Angoa P. M. Francescutti-Verbeem D. M. Shah M. M. Kuhn D. M. (2010). The role of endogenous serotonin in methamphetamine-induced neurotoxicity to dopamine nerve endings of the striatum. J. Neurochem. 115 (3), 595–605. 10.1111/j.1471-4159.2010.06950.x 20722968 PMC2974310

[B73] Veerasakul S. Watiktinkorn P. Thanoi S. Dalton C. F. Fachim H. A. Nudmamud-Thanoi S. (2017). Increased DNA methylation in the parvalbumin gene promoter is associated with methamphetamine dependence. Pharmacogenomics 18 (14), 1317–1322. 10.2217/pgs-2016-0188 28835159

[B74] Villasenor R. Baubec T. (2021). Regulatory mechanisms governing chromatin organization and function. Curr. Opin. Cell Biol. 70, 10–17. 10.1016/j.ceb.2020.10.015 33276273

[B75] Wang C. Kang X. Zhou L. Chai Z. Wu Q. Huang R. (2018). Synaptotagmin-11 is a critical mediator of parkin-linked neurotoxicity and Parkinson's disease-like pathology. Nat. Commun. 9 (1), 81. 10.1038/s41467-017-02593-y 29311685 PMC5758517

[B76] Wang H. Dong X. Awan M. Bai J. (2022). Epigenetic mechanisms involved in methamphetamine addiction. Front. Pharmacol. 13, 984997. 10.3389/fphar.2022.984997 36091781 PMC9458865

[B77] Wang Y. Wei T. Zhao W. Ren Z. Wang Y. Zhou Y. (2021). MicroRNA-181a is involved in methamphetamine addiction through the ERAD pathway. Front. Mol. Neurosci. 14, 667725. 10.3389/fnmol.2021.667725 34025353 PMC8137846

[B78] Werner C. T. Altshuler R. D. Shaham Y. Li X. (2021). Epigenetic mechanisms in drug relapse. Biol. Psychiatry 89 (4), 331–338. 10.1016/j.biopsych.2020.08.005 33066961 PMC7854851

[B79] Wey H. Y. Gilbert T. M. Zurcher N. R. She A. Bhanot A. Taillon B. D. (2016). Insights into neuroepigenetics through human histone deacetylase PET imaging. Sci. Transl. Med. 8 (351), 351ra106. 10.1126/scitranslmed.aaf7551 PMC5784409 27510902

[B80] Xiao J. Ma Y. Wang X. Wang C. Li M. Liu H. (2022). The vulnerability to methamphetamine dependence and genetics: a case-control study focusing on genetic polymorphisms at chromosomal region 5q31.3. Front. Psychiatry 13, 870322. 10.3389/fpsyt.2022.870322 35669261 PMC9163382

[B81] Xu L. Li L. Chen Q. Huang Y. Chen X. Qiao D. (2023). The role of non-coding RNAs in methamphetamine-induced neurotoxicity. Cell Mol. Neurobiol. 43 (6), 2415–2436. 10.1007/s10571-023-01323-x 36752885 PMC11410138

[B82] Xu X. Ji H. Liu G. Wang Q. Liu H. Shen W. (2016). A significant association between BDNF promoter methylation and the risk of drug addiction. Gene 584 (1), 54–59. 10.1016/j.gene.2016.03.010 26976342

[B83] Yang J. Li L. Hong S. Zhang D. Zhou Y. (2020). Methamphetamine leads to the alterations of microRNA profiles in the nucleus accumbens of rats. Pharm. Biol. 58 (1), 797–805. 10.1080/13880209.2020.1803366 32893733 PMC8641683

[B84] Yap E. L. Pettit N. L. Davis C. P. Nagy M. A. Harmin D. A. Golden E. (2021). Bidirectional perisomatic inhibitory plasticity of a Fos neuronal network. Nature 590 (7844), 115–121. 10.1038/s41586-020-3031-0 33299180 PMC7864877

[B85] Zahniser N. R. Sorkin A. (2009). Trafficking of dopamine transporters in psychostimulant actions. Semin. Cell Dev. Biol. 20 (4), 411–417. 10.1016/j.semcdb.2009.01.004 19560046 PMC3248240

[B86] Zeng R. Pu H. Y. Zhang X. Y. Yao M. L. Sun Q. (2023). Methamphetamine: mechanism of action and Chinese herbal medicine treatment for its addiction. Chin. J. Integr. Med. 29 (7), 665–672. 10.1007/s11655-023-3635-y 37074617

[B87] Zhang C. Zhao X. Wang H. J. Yue X. (2021). Research progress on the omics of methamphetamine toxic damage and addiction. Fa Yi Xue Za Zhi 37 (6), 776–787. 10.12116/j.issn.1004-5619.2021.310201 35243842

[B88] Zhang P. Wu W. Chen Q. Chen M. (2019). Non-coding RNAs and their integrated networks. J. Integr. Bioinform 16 (3), 20190027. 10.1515/jib-2019-0027 31301674 PMC6798851

[B89] Zhao Y. Qin F. Han S. Li S. Zhao Y. Wang H. (2022). MicroRNAs in drug addiction: current status and future perspectives. Pharmacol. Ther. 236, 108215. 10.1016/j.pharmthera.2022.108215 35609719

[B90] Zhu L. Li J. Dong N. Guan F. Liu Y. Ma D. (2016). mRNA changes in nucleus accumbens related to methamphetamine addiction in mice. Sci. Rep. 6, 36993. 10.1038/srep36993 27869204 PMC5116666

[B91] Zhu L. Zhu J. Liu Y. Chen Y. Li Y. Huang L. (2015). Methamphetamine induces alterations in the long non-coding RNAs expression profile in the nucleus accumbens of the mouse. BMC Neurosci. 16, 18. 10.1186/s12868-015-0157-3 25884509 PMC4399149

